# Severe gestational diabetes mellitus in lean dams is associated with low IL-1α levels and affects the growth of the juvenile mouse offspring

**DOI:** 10.1038/s41598-023-28903-7

**Published:** 2023-01-30

**Authors:** Lucia Mihalovičová, Veronika Kunšteková, Dávid Miláček, Jakub Janko, Michal Pastorek, Barbora Konečná, Radana Gurecká, Zuzana Rausová, Oľga Uličná, Peter Celec, Katarína Šebeková

**Affiliations:** 1 Institute of Molecular Biomedicine, Faculty of Medicine, Comenius University, Sasinskova 4, 811 08 Bratislava, Slovakia; 2 Department of Biology, Faculty of Medicine, Slovak Medical University, 833 03 Bratislava, Slovakia; 3 Institute of Medical Physics, Biophysics, Informatics and Telemedicine, Faculty of Medicine, Comenius University, 811 08 Bratislava, Slovakia; 4 Pharmacobiochemical Laboratory of 3rd Department of Internal Medicine, Faculty of Medicine, Comenius University, 811 08 Bratislava, Slovakia; 5 Institute of Pathophysiology, Faculty of Medicine, Comenius University, 811 08 Bratislava, Slovakia; 6 Department of Molecular Biology, Faculty of Natural Sciences, Comenius University, 842 15 Bratislava, Slovakia

**Keywords:** Developmental biology, Molecular biology, Physiology, Biomarkers, Molecular medicine

## Abstract

We investigated how maternal gestational diabetes (GDM) impacts the metabolic status of offspring. GDM was induced in CD1 mice consuming a fast-food diet (FFD) by repeated low-dose streptozotocin injections before mating. Offspring of normoglycemic standard chow or the FFD consuming dams served as controls. In 4-week-old offspring weaned to standard chow, plasma concentrations of extracellular DNA, inflammatory markers, and parameters of the cardiometabolic status (glycemia, liver lipid content; body, organ, and fat weight) were determined. Two-factor analysis of variance indicated that the male offspring of GDM dams manifest postnatal growth retardation and lower relative kidney weight. Regardless of sex, GDM offspring manifest the lowest IL-1α levels, and other inflammatory markers showed mild and inconsistent alterations. Offspring of dams consuming the FFD displayed higher liver triacylglycerols content. The three groups of offspring showed no significant differences in glycemia and extracellular DNA. Partial least squares-discriminant analysis indicated that male GDM offspring present lower kidney, body, and brown adipose tissue weights; lower IL-1α levels, and higher concentrations of GM-CSF and IL-10 compared with their FFD counterparts. The model failed to select discriminative variables in females. In conclusion, in mice, maternal GDM in the absence of obesity adversely affects the early growth of juvenile male offspring.

## Introduction

Gestational diabetes mellitus (GDM) is defined as glucose intolerance that first manifests or is first diagnosed during pregnancy^1^. GDM is the most common form of hyperglycemia in pregnancy, accounting for approximately 80–85% of all cases^2^. It affects 14% to 25% of pregnancies worldwide^3,4^. Although overweight and obesity are important risk factors for GDM, approximately half of mothers with GDM are obese^5^. The developmental origins of the health and disease (DoHaD) hypothesis postulates that through developmental plasticity, intrauterine and early postnatal stressors may increase the risk of manifesting chronic diseases later in life^6^. Intrauterine hyperglycemia per se as well as several GDM-associated conditions may lead to an increased risk of the offspring developing obesity, glucose intolerance, or type 2 diabetes later in life^7,8^. In this context, multiple factors have been implicated in unfavorable health outcomes for mothers and children.

There is a positive regulatory loop between hyperglycemia and metainflammation, in which hyperglycemia induces the placental and peripheral synthesis of proinflammatory cytokines, chemokines, and adipokines^9–11^. Then, an inflammation triggers the formation of neutrophil extracellular traps (NETs), and the placenta secretes extracellular vesicles and extracellular deoxyribonucleic acids (ecDNA)^11–14^. This process may play a role in this condition. EcDNA of nuclear (ncDNA) and mitochondrial (mtDNA) origin is released into body fluids during cell death, e.g., necrosis, apoptosis, and NETosis^14,15^. This is a danger signal (a damage-associated molecular pattern) recognized by toll-like receptor 9, which leads to the production of inflammatory cytokines and chemokines^16^. To maintain low ecDNA levels, the organism degrades ecDNA by deoxyribonucleases (DNases)^17^. In addition to its pathophysiological role in preeclampsia, ecDNA is implicated in obesity^18,19^, and obesity-associated insulin resistance^20^. The higher the plasma ecDNA concentrations and the mitochondrial DNA (mtDNA) copy number are, the higher the cardiometabolic risk^21^. Low ecDNA levels were recently suggested as novel markers for neonatal well-being^22^.

As GDM is the most common complication of pregnancy, experimental rodent models to study the pathogenetic mechanisms of GDM and their contribution to adverse health outcomes have been introduced^23,24^. Nutritional models mostly mimic obesity-associated GDM, which is achieved by the administration of different obesogenic diets (generally high-fat diet (HFD) or high-fat/high-sugar diet) either pre-conceptionally, before parturition, or even until the weaning of offspring. Streptozotocin (STZ) is the most common chemical to induce GDM and allows for the induction of GDM in the absence of maternal obesity. It is applied to dams in different dosages; ways and frequencies of administration; with timings of administration ranging between Day 1 and mid-term of pregnancy^23,24^. As STZ can cross the placenta, its administration in the early pregnancy might affect embryonic development^25,26^. However, if STZ is administered later in gestation, it might affect the developing fetal β-cells^24,27^. Offspring early outcomes differ. Studies in which maternal obesity was achieved report either fetal macrosomia and higher body weight in weanlings^28–30^, no weight difference or even growth restriction^31,32^. However, HFD-induced lean maternal GDM does not affect the body weight of the pups^33^. Mild maternal STZ-induced hyperglycemia was associated with fetal macrosomia^34,35^, while dams with severe GDM had offspring with low body weights^35–37^. Despite the recommendations of the National Institute of Health (NIH) to consider sex in the design and interpretation of preclinical studies^38^, sex differences are seldom communicated. Even if they are, negative effects are reported for male offspring^30,32,34^.

In this study, we induced marked gestation-specific hyperglycemia in dams by combining fast-food diet (FFD)^39^ feeding (preconceptionally until parturition) with repeated low-dose STZ treatments before pregnancy. Offspring of normoglycemic mice consuming an FFD or standard chow served as control groups. All offspring were weaned onto a standard diet. Except for indicators of growth and metabolic status, we focused on plasma ecDNA as it remains unclear whether an intrauterine hyperglycemic milieu affects ecDNA levels and DNase activity in juvenile offspring^32^ and whether concentrations of anti/proinflammatory cytokines potentially induced by ecDNA act as a damage-associated molecular pattern. As offspring of GDM mothers have an increased risk for metabolic and cardiovascular complications^40,41^, we hypothesized that the offspring of GDM dams will present adiposity and higher levels of glucose, circulating ecDNA, and inflammatory markers than offspring of normoglycemic dams. We focused on sex differences since evidence suggests that male and female offspring respond differently to preconceptional and in utero environment programming the intergenerational inheritance of metabolic traits^42^.

## Results

### The FFD did not influence the body weight of the dams

**Figure 1:**
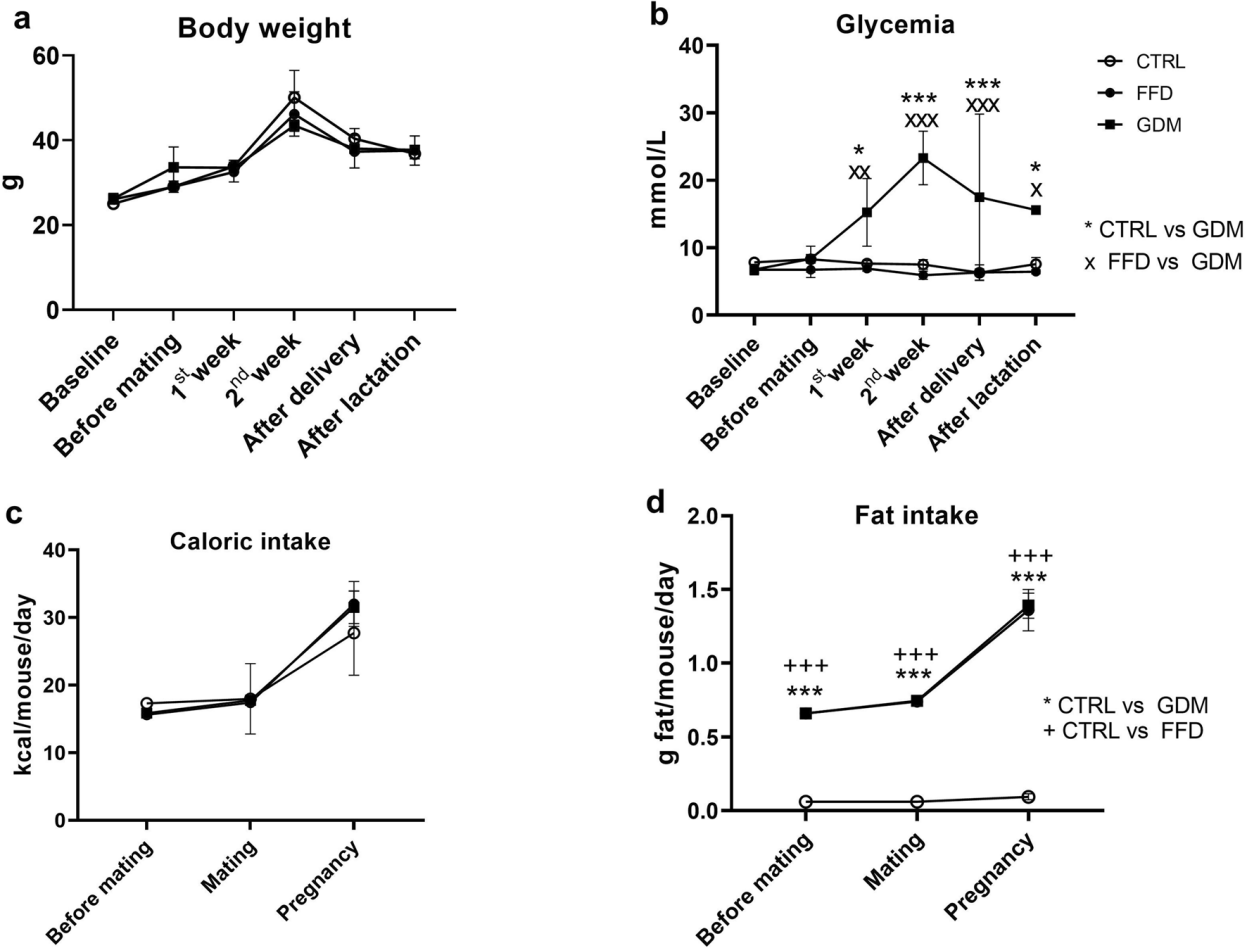
(**a**) Time-course of body weight in dams: p_group_ = 0.808, p_time_ < 0.001, p_group*time interaction_ = 0.107; (**b**) time course of random glycemia in dams: p_group_ = 0.013, p_time_ = 0.045, p_group*time interaction_ < 0.001; (**c**) mean daily caloric intake of dams before mating, during mating and pregnancy: p_group_ = 0.883, p_time_ < 0.001, p_group*time interaction_ = 0.540; (**d**) mean daily fat intake of dams before mating, during mating and pregnancy: p_group_ < 0.001, p_time_ < 0.001, p_group*time interaction_ < 0.001; groups: *CTRL* dams consuming control diet, *FFD* dams consuming fast-food diet (cheeseburgers), *GDM* dams with gestational diabetes mellitus. *^,+^p < 0.05, **^,++^p < 0.01, ***^,+++^p < 0.001.

One dam from the GDM group did not conceive and thus was excluded from the analyses. Throughout the study, a significant time impact on body weight was present (Fig. 1a; p < 0.001), but no between-group differences were observed. From the 1st week of pregnancy, random glycemia was higher in GDM females than in CTRL and FFD dams (15.3 ± 5.02 mmol/L vs. 7.7 ± 0.4 mmol/L and 6.9 ± 0.4 mmol/L, respectively). In dams with GDM, glycemia peaked during the second week (23.3 ± 4.0 mmol/L) and remained elevated even after the suckling period. However, the CTRL and FFD dams presented normal random glycemia (2nd week: 7.5 ± 0.7 mmol/L and 5.9 ± 0.6 mmol/L, respectively) (Fig. 1b). Caloric intake did not differ between groups throughout the experiment (Fig. 1c), although FFD and GDM dams consumed significantly more fat than their CTRL counterparts (Fig. 1d; p < 0.001 both).

### Male offspring of GDM dams showed growth retardation

**Figure 2:**
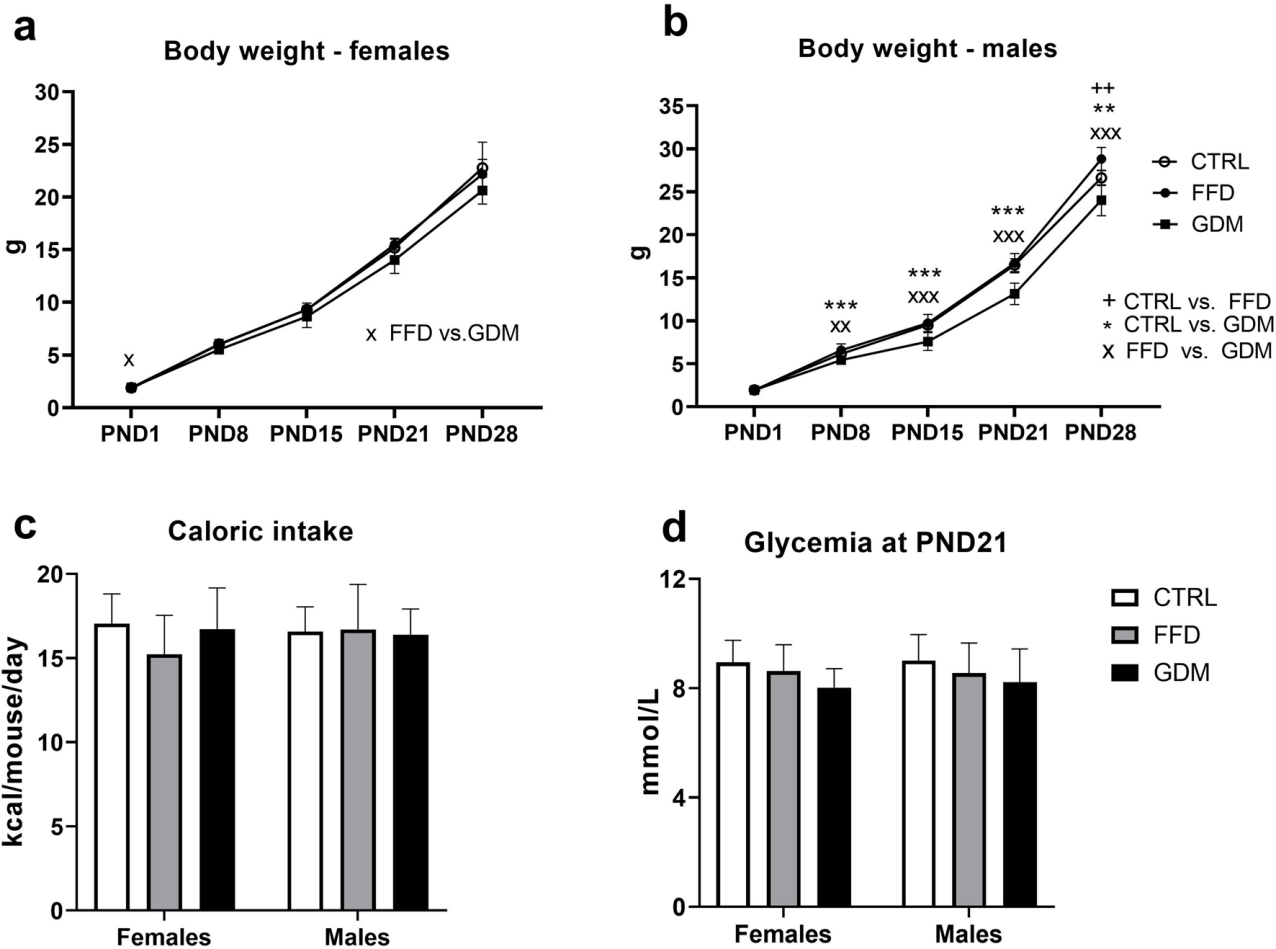
(**a**) Body weight curve in female offspring: p_group_ = 0.015, p_time_ < 0.001, p_group*time interaction_ = 0.014; (**b**) body weight curve of male offspring: p_group_ < 0.001, p_time_ < 0.001, p_group*time interaction_ < 0.001; (**c**) mean caloric intake per day: p_group_ = 0.688, p_sex_ = 0.356, p_group*sex interaction_ = 0.224; (**d**) glycemia: p_group_ = 0.043, p_sex_ = 0.810, p_group*sex interaction_ = 0.922; groups: *CTRL *offspring of dams consuming control diet, *FFD* offspring of dams administered fast-food diet (cheeseburgers), *GDM* offspring of dams with gestational diabetes mellitus; ^x^p < 0.05; **^,xx,++^p < 0.01; ***^,xxx^p < 0.001.

At postnatal day 1 (PND1), female offspring of FFD dams were slightly but significantly lighter compared with the female GDM offspring (1.89 ± 0.12 vs. 1.94 ± 0.07 g, respectively); later in the study, no significant differences in the body weights of female offspring were observed (Fig. 2a). Three groups of male offspring displayed similar body weights at birth. From PND8 until the end of the study, the offspring of GDM dams were lighter than the offspring of CTRL and FFD dams (Fig. 2b; p < 0.001; both). At sacrifice (PND28), FFD males reached the highest weight, followed by CTRL and GDM counterparts. During the postweaning week, caloric intake did not differ significantly between the groups (either males or females; Fig. 2c). At weaning (PND21; Fig. 2d), GDM offspring displayed mildly lower random glycemia than the CTRL and FFD offspring, but the Sidak’s post-hoc test failed to localize between group significance within the sexes.

### The relative weight of the perirenal fat or heart did not differ significantly between the groups (Table 1)

**Table 1:** Relative organ weight and biochemical data of the offspring.

	Males						Females						p-group	p-sex	p-g*s
	CTRL (N = 11)		FFD (N = 9)		GDM (N = 11)		CTRL (N = 13)		FFD (N = 15)		GDM (N = 5)				
	M	± SD	M	± SD	M	± SD	M	± SD	M	± SD	M	± SD			
PRF (%)	0.12	0.05	0.15	0.07	0.12	0.03	0.13	0.08	0.08	0.04	0.10	0.03	0.428	0.085	**0.039**
Liver (%)	6.33	0.26	6.47	0.65	6.58	0.48	5.69	0.40	5.35	0.50	5.93	0.43	0.099	**< 0.001**	0.162
Spleen (%)	0.38	0.07	0.34	0.05	0.39	0.10	0.48	0.20	0.39	0.05	0.40	0.09	0.141	0.083	0.444
Heart (%)	0.55	0.04	0.55	0.04	0.52	0.04	0.55	0.09	0.53	0.07	0.50	0.04	0.165	0.510	0.965
AOPP (μmol/L)	276	121	300	121	253	114	223	87	253	89	240	76	0.565	0.158	0.817

Significant values are in bold.

*CTRL* offspring of dams consuming a control diet, *FFD* offspring of dams administered a fast-food diet (cheeseburgers), *GDM* offspring of dams with gestational diabetes mellitus, g*s group-sex interaction, *PRF* perirenal fat, *AOPP* advanced oxidation protein products; organ and fat-pad weights are expressed as a percent of body weight, *M* mean, *SD* standard deviation, *N* number of animals.

Similarly, the relative weights of uteri (p = 0.659) or testes (p = 0.058) did not show between-group differences. Male offspring showed a higher relative liver weight than females (p < 0.01). Plasma advanced oxidation protein product (AOPP) concentrations did not differ significantly between the groups (Table 1). Due to the low volumes of obtained plasma, creatinine, high-density lipoprotein (HDL-C), low-density lipoprotein cholesterol (LDL-C) concentrations, and liver enzyme activities were analyzed only in 39 animals (data are given in Supplementary Table 1).

### The relative weight of the brown adipose tissue (BAT) declined across the three groups in both sexes but significance was reached only for GDM vs. CTRL male offspring (Fig. 3a)

**Figure 3:**
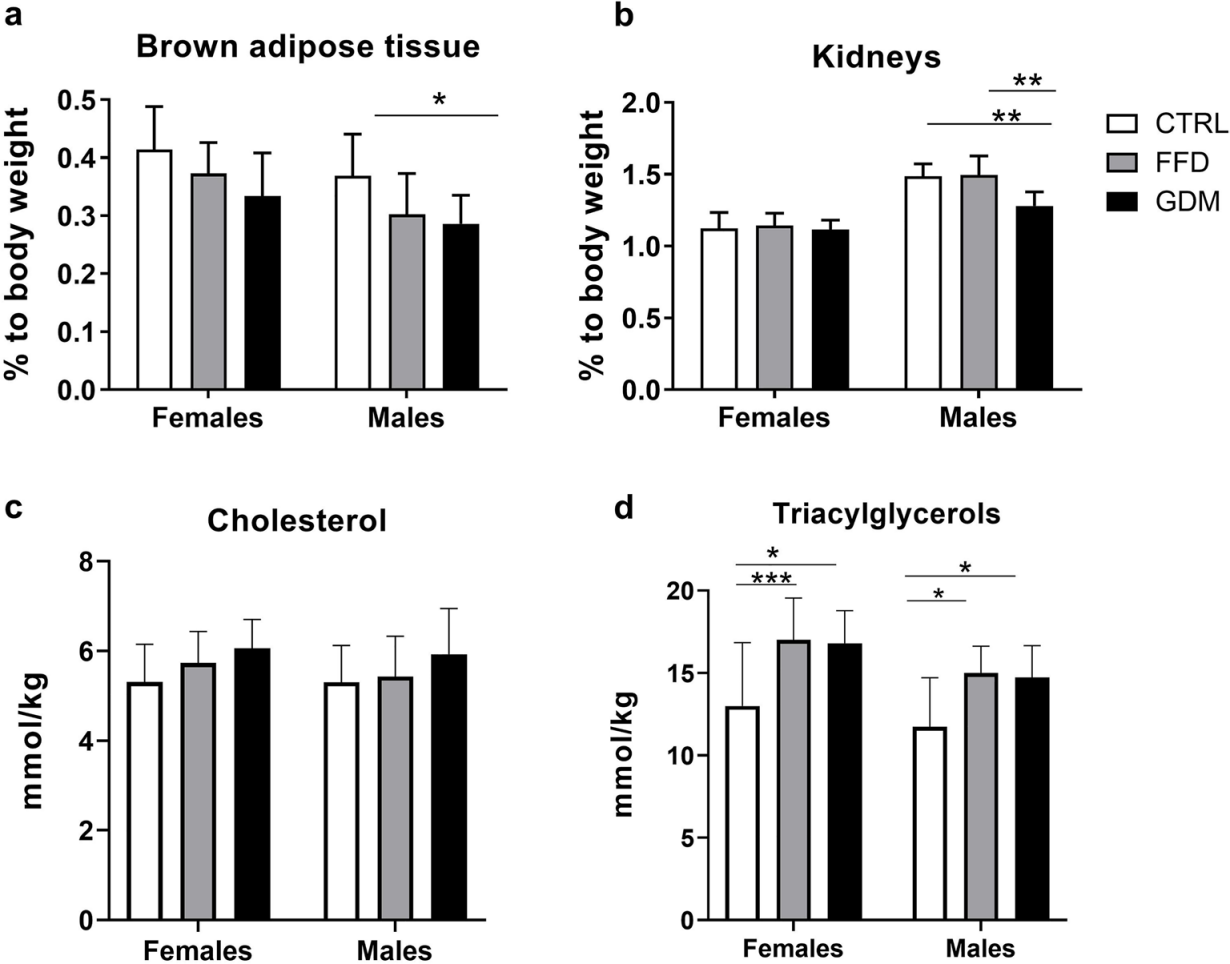
The relative weights of the (a) brown adipose tissue: p_group_ = 0.001, p_sex_ = 0.003, p_group*sex interaction_ = 0.792, and (b) kidneys: p_group_ = 0.002, p_sex_ < 0.001, p_group*sex interaction_ = 0.009; (c) liver content of total cholesterol: p_group_ = 0.601, p_sex_ = 0.514, p_group*sex interaction_ = 0.834; (d) liver content of triacylglycerols: p_group_ < 0.001, p_sex_ = 0.016, p_group*sex interaction_ = 0.862; groups: *CTRL* offspring of dams consuming control diet, *FFD* offspring of dams administered fast-food diet (cheeseburgers), *GDM* offspring of dams with gestational diabetes mellitus; *p < 0.05; **p < 0.01; ***p < 0.001.

Moreover, males displayed significantly lower BAT/body weight than females. Male offspring showed a higher relative weight of the kidneys than females (Fig. 3b). Kidney/body weight was lower in GDM male offspring than in both the CTRL and FFD groups (p < 0.01). Liver cholesterol content showed an increasing trend across the three categories in both sexes, but significance was not reached (Fig. 3c). Females displayed a higher liver content of triacylglycerols (TAG) than males, and in both sexes, the offspring of FFD and GDM dams showed a higher accumulation of TAG than the controls (Fig. 3d).

### Levels of ecDNA were not affected by maternal intrauterine factors

**Figure 4:**
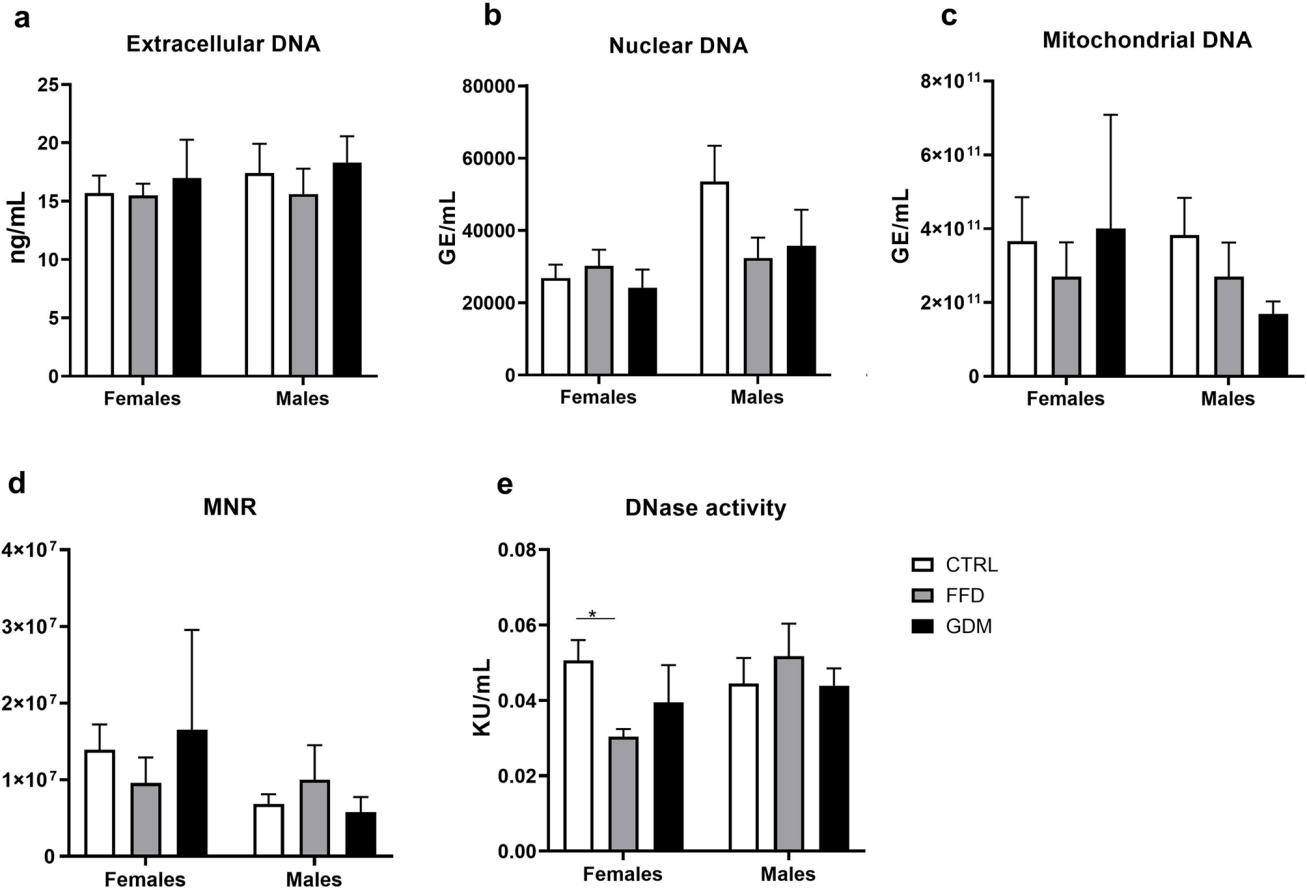
Data from the offspring. (**a**) Plasma extracellular deoxyribonucleic acid concentrations: p_group_ = 0.631, p_sex_ = 0.537, p_group*sex interaction_ = 0.916; (**b**) numbers of nuclear genomic equivalents (GE) in plasma: p_group_ = 0.302, p_sex_ = 0.033, p_group*sex interaction_ = 0.215; (**c**) numbers of mitochondrial genomic equivalents (GE) in plasma: p_group_ = 0.625, p_sex_ = 0.486, p_group*sex interaction_ = 0.576; (**d**) ratio of mitochondrial-to-nuclear (MNR) genomic equivalents: p_group_ = 0.957, p_sex_ = 0.104, p_group*sex interaction_ = 0.449; (**e**) deoxyribonuclease (DNase) activity in plasma: p_group_ = 0.438, p_sex_ = 0.187, p_group*sex interaction_ = 0.048; *KU* Kunitz units; groups: *CTRL* offspring of dams consuming control diet, *FFD* offspring of dams administered fast-food diet (cheeseburgers), *GDM* offspring of dams with gestational diabetes mellitus; *p < 0.05.

Neither total plasma ecDNA (Fig. 4a) nor its components—ncDNA (Fig. 4b), mtDNA (Fig. 4c), or the mtDNA-to-ncDNA ratio (Fig. 4d) differed significantly between the offspring groups. Deoxyribonuclease activity was higher in CTRL females than in the FFD group (Fig. 4e; p < 0.05). As short ncDNA and mtDNA fragments could have escaped replication in the PCR assay, ecDNA fragmentation analysis was performed on pooled DNA samples from the offspring of each dam. Specific fragments of a certain length were not revealed (Supplementary Fig. S1).

### GDM offspring displayed the lowest IL-1α concentrations

**Figure 5:**
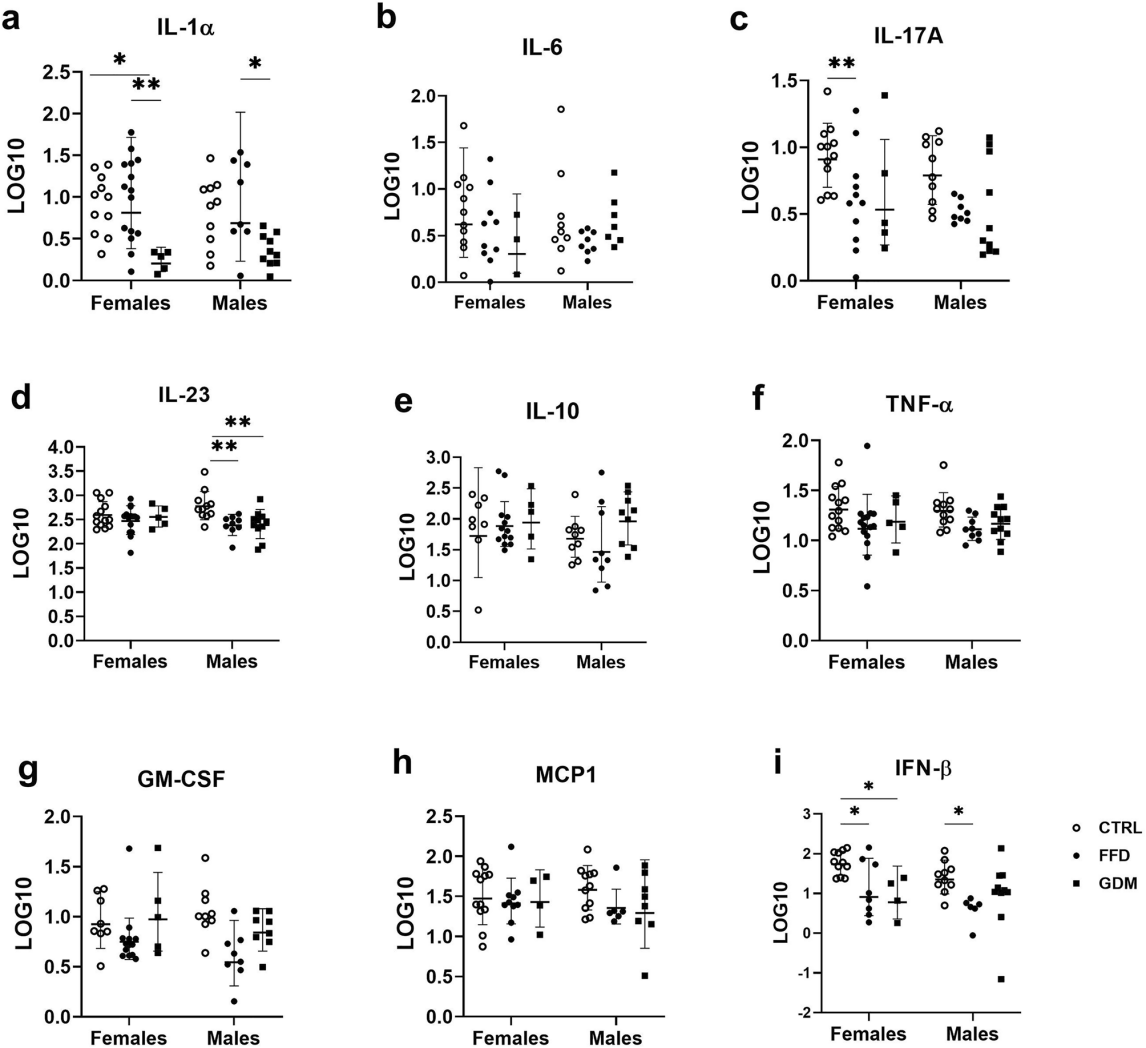
Inflammatory markers in the offspring. (**a**) Interleukine-1 alpha: p_group_ < 0.001, p_sex_ = 0.884, p_diet*sex interaction_ = 0.866; (**b**) interleukine-6: p_group_ = 0.066, p_sex_ = 0.716, p_group*sex interaction_ = 0.704; (**c**) interleukine-17A: p_group_ < 0.001, p_sex_ = 0.395, p_group*sex interaction_ = 0.603; (**d**) interleukine-23: p_group_ = 0.005, p_sex_ = 0.739, p_group*sex interaction_ = 0.113; (**e**) interleukine-10: p_group_ = 0.498, p_sex_ = 0.344, p_group*sex interaction_ = 0.416; (**f**) tumor necrosis factor-alpha: p_group_ = 0.019, p_sex_ = 0.626, p_group*sex interaction_ = 0.992; (**g**) granulocyte–macrophage colony-stimulating factor: p_group_ = 0.030, p_sex_ = 0.197, p_group*sex interaction_ = 0.717; (**h**) monocyte chemoattractant protein-1: p_group_ = 0.293, p_sex_ = 0.824, p_group*sex interaction_ = 0.673; (**i**) interferon-beta; p_group_ < 0.001, p_sex_ = 0.107, p_group*sex interaction_ = 0.468; *p < 0.05, **p < 0.01, ***p < 0.001; groups: *CTRL* offspring of dams consuming control diet, *FFD* offspring of dams administered fast-food diet (cheeseburgers), *GDM* offspring of dams with gestational diabetes mellitus, *Log10* decadic logarithm, markers are given in pg/mL.

Circulating levels of pro- and anti-inflammatory markers are given in Fig. 5a–i. IL-1α concentrations were lower in the GDM female offspring than in both other groups; these concentrations were also lower in GDM males than in FFD males (Fig. 5a). In females, the CTRL group displayed higher IL-17A concentrations than the FFD group (Fig. 5c; p < 0.01). Among males, IL-23 levels were higher in the offspring of the CTRL dams than in FFD and GDM counterparts (Fig. 5d; p < 0.01; both). INF-β concentrations were higher in CTRL offspring, than in both other groups in females and vs. the offspring of FFD dams in males (Fig. 5i; p < 0.05; all). No significant effect of sex or the group*sex interaction was revealed for either variable.

### Multivariate analyses

**Figure 6:**
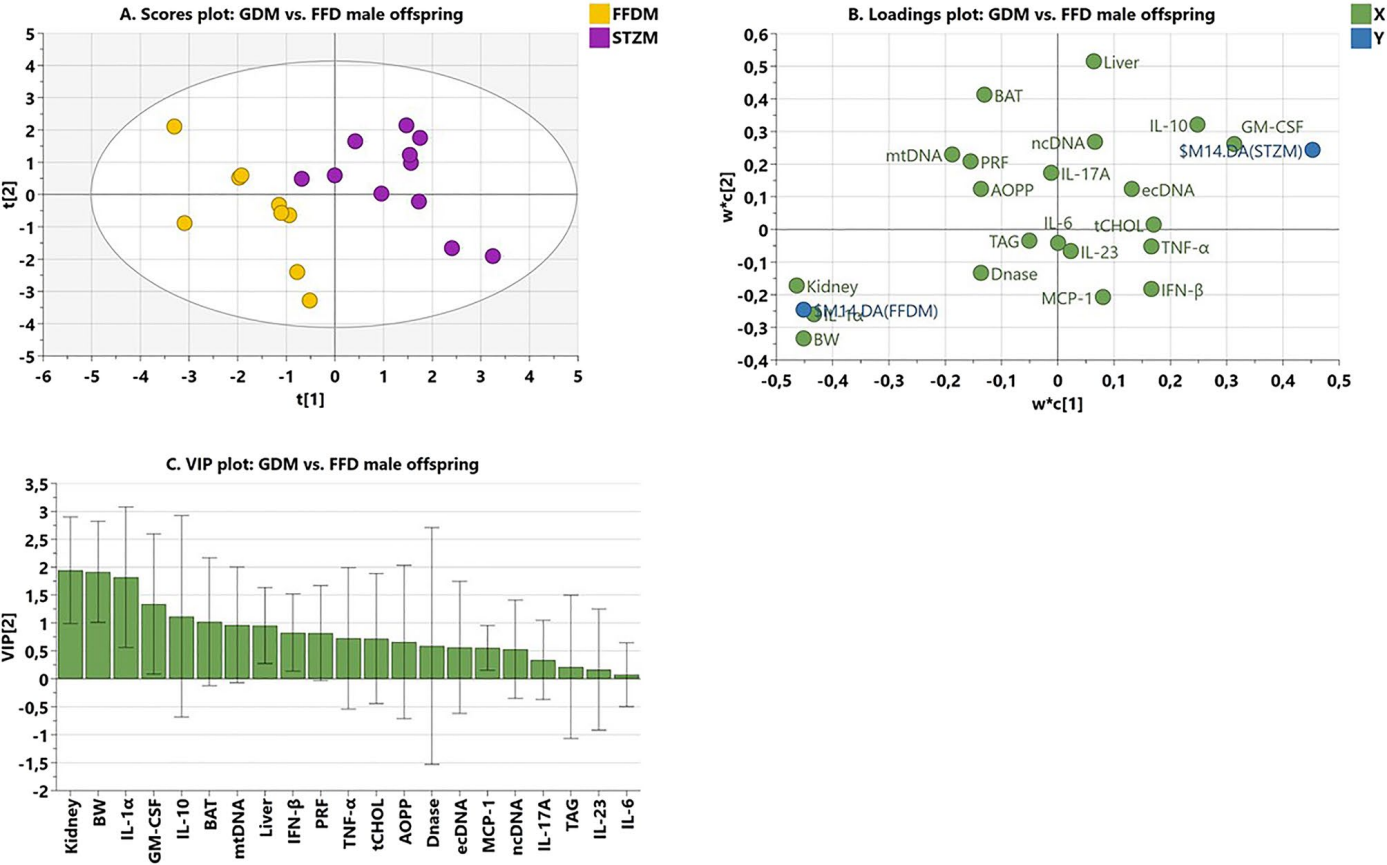
(**A**) The scatter plot of the scores visualizes in the horizontal direction the separation between the male offspring of dams consuming the fast-food diet (FFD, yellow circles) and those from dams consuming an FFD presenting with gestational diabetes mellitus (STZ, violet circles); while the vertical direction shows the within-group variability; (**B**) loading scatter plot displays the relationship between independent variables (green circles) and dummy variables representing the groups of FFD and STZ (GDM) male offspring (blue circles). Independent variables situated in the vicinity of dummy variables have the highest discriminatory power among the groups; those positioned near the FFD dummy variable are higher in FFD offspring; while those nearby the STZ (GDM) dummy variable were higher in GDM than in FFD offspring; (**C**) the VIP plot summarizes the importance of the variables for between-group separation sorted from high to low. Variables with VIP ≥ 1.00 are considered significant. BW, body weight; kidney, liver, BAT, PRF relative weight of kidneys, liver, brown adipose tissue, and perirenal fat, respectively*; Chol, TAG* liver content of total cholesterol and triacylglycerols, respectively; *ecDNA* the concentration of extracellular DNA; *ncDNA, mtDNA* genomic equivalents of nuclear and mitochondrial DNA in plasma, respectively; *IL-1α* interleukine-1 alpha; *IL-6* interleukine-6; *IL-17A* interleukine-17A; *IL-23* interleukine-23; *IL-10* interleukine-10; *TNF-α* tumor necrosis factor-alpha; *GM-CSF* granulocyte–macrophage colony-stimulating factor; *MCP-1* monocyte chemoattractant protein-1; *INF-β* interferon-beta.

To reveal the variables with the largest discriminant power between the FFD and GDM groups of offspring, the partial least squares-discriminant analysis (PLS-DA) model was used. Before running the PLS-DA, we checked for potential outliers using the principal component analysis (PCA) model. The PCA indicated that in males all scores were situated within Hotelling’s T2 tolerance ellipse. The scatter plot (Fig. 6A) showed that the groups were well separated and did not overlap (FFD male offspring: yellow circles; their GDM counterparts: violet circles). The loading plot (Fig. 6B) shows the relationship between the independent variables (green circles) and dependent variables, e.g. dummy variables indicating the groups of FFD and STZ offspring (blue circles). Independent variables that express the between-group difference are positioned in the vicinity of dummy variables. The variable of importance for the projection (VIP plot; Fig. 6C) summarizes the importance of the variables for between-group separation sorted from high to low. The model identified relative kidney weight, body weight, the levels of IL-1α, GM-CSF, and IL-10, and the relative weight of BAT (VIP 1.94 to 1.02) as major variables contributing to the between-group difference. As shown in the loading plot (Fig. 6B), GDM offspring presented lower relative kidney weight, body weight, IL-1α levels and the relative weight of BAT and higher GM-CSF and IL-10 levels than their FFD counterparts. Moreover, the grouping of independent variables suggests their interrelationship, confirmed by Pearson’s correlations (relative kidney weight to bodyweight: r = 0.473, p < 0.05; log IL-1α to body weight: r = 0.559, p < 0.01; log IL-1α to relative kidney weight: r = 0.626, p < 0.01; log IL-10 to GM-CSF: 0.519, p < 0.05), while the relative weight of BAT correlated with log IL-1α (r = 0.504, p < 0.05). The variation explained by the model was satisfactory (R^2^ = 82%), while its ability to predict new data was low (Q^2^ = 37%). These findings probably reflect the great interindividual variability in IL-10 levels, the relative weight of BAT, and GM-CSF values (Fig. 6C).

For significant predictors selected by the PLS-DA model, we checked for post-hoc power. At α ≤ 0.05, the test indicated a power of 98% for the body weight and relative kidney weight, 93% for IL-1α levels, and 69% for GM-CSF, while for IL-10 levels and relative BAT weight, it was ≤ 40%.

In female offspring, PCA indicated a major outlier. Even after its exclusion, the PLS-DA failed to model the between-group difference. The goal was not achieved probably due to the low number of GDM female offspring and the high variability of the data.

## Discussion

We investigated whether the juvenile offspring of nonobese dams with GDM that consumed an FFD preconceptionally and during pregnancy present altered growth, metabolic and inflammatory statuses compared with their counterparts from normoglycemic dams consuming either standard chow or an FFD. The offspring neither came into direct contact with STZ nor with an FFD. As expected, dams in the GDM group but not the other two groups manifested gestational hyperglycemia. The 2-factor ANOVA indicated that the male offspring of GDM dams manifested postnatal growth retardation and lower relative kidney weights compared with male offspring in the other two groups. Regardless of sex, the offspring of dams consuming the FFD showed a higher accumulation of triacylglycerols in the liver. Plasma ecDNA concentrations did not differ significantly among the groups. Those of inflammatory markers seldom showed significant differences and, if so, were generally slightly but significantly higher in the offspring of CTRL dams. However, in the multivariate analysis, male offspring from GDM dams differed from their FFD counterparts in the variables characterizing body frame, and they also presented with lower IL-1α levels.

Cheeseburgers were used to model a human FFD. This is a diet high in saturated fats, as approximately 38% of energy is provided from fats. In comparison, rodent high-fat diets used for induction of GDM provide 50–60% energy from fats—generally lard. By fat-derived energy content as well as composition, these diets differ profoundly from the high-fat diets of humans. The lower energy content of cheeseburgers compared with standard rodent chow stems from their higher water content (49%), as calorically dense pellets have a low water content (8–12%)^43,44^. Mice effectively compensated for the lower caloric content of cheeseburgers by their higher consumption. The mean caloric intake of the three groups of dams was similar, although the FFD groups consumed substantially more fats. In contrast, female CD1 mice rapidly gained weight on a HFD with 60% fat content despite similar caloric intake to those consuming chow^45^. Our dams did not gain excess weight despite consuming a FFD providing more metabolizable calories from fat. It cannot be said for sure whether the difference is due to the content or composition of the fat.

Dams on the standard diet or the FFD remained normoglycemic throughout the study. None of them displayed random blood glucose levels ≥ 12 mmol/L, which has been proposed as a diagnostic threshold for hyperglycemia in wild-type mice^46^. Those administered STZ developed severe gestational hyperglycemia with a decreasing trend after delivery and lactation. Lean rodents with severe gestational hyperglycemia deliver offspring with lower birth weights compared with pups of normoglycemic dams, and the offspring further show an impaired postnatal growth rate^36,37^. In our study, maternal GDM did not affect the birth weight of male pups. However, these offspring showed growth retardation from PND8 until sacrifice, which was associated with a lower relative kidney weight. The offspring of mice with GDM displayed low body weights, small kidneys with smaller glomeruli, and a low number of nephrons^37^. Brenner and McKenzie hypothesize that newborns with fewer number of nephrons are at an increased risk for hypertension and end-stage renal disease in adulthood^47,48^. Similar to humans, nephrogenesis is also completed before birth in mice^49^. Thus, mouse models of an intrauterine hyperglycemic environment may shed light on the potential renal consequences in offspring later in life.

In contrast to our hypothesis, the GDM offspring neither developed adiposity nor manifested impaired glycemia. Females even presented with lower relative amounts of perirenal fat. The amounts of brown adipose tissue was the lowest in the GDM offspring of both sexes and lower in males than females. Sex differences in the size and function of BAT appear to be female biased across all animal species, and BAT has been proposed as an indicator of metabolic health^50^. Regarding glycemia, our outcomes correspond with those observed in juvenile offspring of dams with severe maternal STZ-induced hyperglycemia^36^. In different studies, adolescent offspring of lean dams with GDM exhibited higher adiposity with normal fasting insulinemia and glucose tolerance^32^, while those from obese dams with diet-induced GDM presented at four weeks of age with higher body weights and fasting glycemia compared with their counterparts from lean normoglycemic dams. However, only male offspring displayed insulin resistance^30^. Thus, depending on the experimental conditions, fetal exposure to hyperglycemia manifests in juvenile rodent offspring with varying phenotypes for adiposity, glucose tolerance, and insulin sensitivity.

There were no significant differences in hepatic cholesterol content levels between the sexes. We only observed a tendency toward an increase across the three maternal dietary groups, with the highest levels in GDM offspring, but without reaching statistical significance. However, a study in mice showed that maternal consumption of a HFD (60% energy from fats) preconceptionally led to increased expression of genes and proteins required for de novo cholesterol synthesis in the liver of male offspring administered a HFD for 12 weeks after weaning^51^. To our knowledge, the effect of GDM on offspring liver cholesterol content has not been reported. Moreover, our offspring of dams consuming FFD (regardless of whether they manifested GDM) showed a similar accumulation of TAG in the liver, which was higher than that in their CTRL counterparts. Females displayed a higher liver TAG content than males. In other studies, male offspring but not female offspring of dams with HFD- or STZ-induced GDM displayed ectopic deposition of lipids in livers^30,31^. The maternal HFD-induced dysmetabolic liver phenotype demonstrates sex differences. Female offspring are prone to liver steatosis and nonalcoholic fatty liver disease; males rather develop liver fibrosis and manifest nonalcoholic steatohepatitis^52^. The higher TAG accumulation in the livers of our female vs. male FFD and GDM offspring mirrors this observation. Thus, both a maternal HFD, as well as intrauterine hyperglycemia, present a threat to the liver health of future generations.

In contrast to our hypothesis, we revealed no differences in ecDNA concentrations among the three groups of offspring. The half-life of ecDNA in circulation is short, approximately minutes, as DNase I effectively cleaves ecDNA^53^. Under physiological conditions, NETosis is one of the major sources of myeloperoxidase and ecDNA in plasma^54^. AOPPs are formed exclusively via the myeloperoxidase reaction, particularly on albumin^55^ which has a plasma half-life approximately three weeks. Thus, similar concentrations of AOPPs in the offspring do not support the assumption of major fluctuations in ecDNA production via NETosis throughout the lifespan of our offspring. Whether similar ecDNA levels and ncDNA, and mtDNA copy numbers in our control, FFD, and GDM offspring might testify to good health remains unclear. In humans, both maternal and fetal complications are associated with an increase in ecDNA in newborns^22^. This small study reported that GDM did not significantly affect ecDNA levels in newborns. However, the subgroup of mothers presenting with GDM was small, and GDM was well controlled.

Maternal GDM is related to systemic and placental inflammatory processes^56,57^, but less is known about how in utero exposure to GDM alters the immune microenvironment of offspring. We did not confirm our hypothesis on the manifestation of low-grade inflammation by GDM offspring, albeit both GDM and HFD consumption may affect cytokine status^46,58,59^. In our animals, cytokine/chemokine levels were within physiological ranges. In the few cases when Sidak’s test indicated an independent impact of maternal GDM, cytokine/chemokine levels were the highest in the control offspring. A missing inflammatory response might reflect the fact that the offspring did not manifest obesity. Obesity predisposes individuals to a proinflammatory state via the increased secretion of inflammatory mediators, particularly IL-6, MCP-1, and TNF-α, from adipose tissue^60,61^. Although obesity-induced DNA released from adipocytes stimulates chronic adipose tissue inflammation^18,19^, we did not reveal an association between ecDNA and inflammatory status in offspring. IL-1α was the single cytokine independently affected by maternal GDM in both sexes. IL-1α is involved in hematopoiesis and the regulation of inflammatory processes. Data on IL-1α in pregnancies or its response to HFD are scarce. A study on healthy and GDM pregnancies did not reveal significant differences in IL-1α levels in umbilical cord blood^46^. Maternal consumption of a HFD did not significantly affect serum IL-1α concentrations in male mouse offspring^62^. However, HFD-induced obesity was associated with higher plasma IL-1α levels, and IL-1α deficiency reduced adiposity, glucose intolerance, and hepatic lipogenesis in HFD-induced obesity in mice^58^. We speculate that lower IL-1α levels in our GDM offspring might have exerted protection against the manifestation of insulin resistance and the development of adiposity.

The low number of dams appears to be the main limitation of our study. However, some studies employed a similar number of dams as ours, and we also employed the same reduction in the number of pups per litter to minimize between- and intralitter variability^59,63^. The design of our study does not allow for the discrimination of whether the discrepancy between the results returned by 2-factor ANOVA and PLS-DA is purely statistical bias or whether multivariate analysis data potentially reflect a pattern that might be of biological impact. ANOVA is a linear model comparing the means. The PCA model converts each individual into a dummy variable based on all centered independent components entered into the model and computes the principal components by which the groups differ. Moreover, it is robust against nonnormally distributed and partially intercorrelated data. The advantage of our study is the fact that the model mimics human GDM pregnancy, and the offspring did not come into contact with either toxic STZ or the FFD. Determination of ecDNA and a panel of cytokines in juvenile offspring is a novelty. In line with the policy of the NIH on preclinical research, we considered sex as a biological variable.

Early life programming generally causes a well-defined pathological phenotype rather later in life than shortly after birth or in young individuals. Sex differences inherited intergenerationally are affected not only by sex chromosomes and hormones but also via epigenetic mechanisms. These mechanisms can modify programming during germline development, leading to sex-related differences in gene expression^42^. A challenge for future research is unveiling whether and how early alterations induced by maternal GDM persist or even evolve in offspring with aging. Additionally, how postnatal exposure to positive or negative environmental challenges (e.g., physical activity or HFD, respectively) affects the phenotype of GDM offspring in adulthood and the mechanisms behind the observed early alterations and those with potential onset in later life remain elusive. Knowledge of which early detectable changes or their cluster are predictive for the later manifestation of cardiometabolic afflictions in GDM offspring is important for further experimental research focused on prevention or therapy. Additionally, such findings may have translational relevance for clinical research and practice.

## Materials and methods

### Animals

Four-week-old female and male CD1 mice (n = 9; each) were purchased from Velaz (Prague, Czech Republic). All mice were housed under controlled conditions (12:12 light/dark cycle, room temperature: 22 ± 2 °C, humidity: 55 ± 10%) with free access to water and standard rodent chow (Eypy-KMK20, Sedliště, Czech Republic)*.* All experimental procedures were approved by The State Veterinary and Food Administration of the Slovak Republic under protocol code 2606/16-221 and were conducted following the EU Directive 2010/63/EU and Slovak legislation. The study is reported following the ARRIVE guidelines.

### Design of the experiment

**Figure 7:**
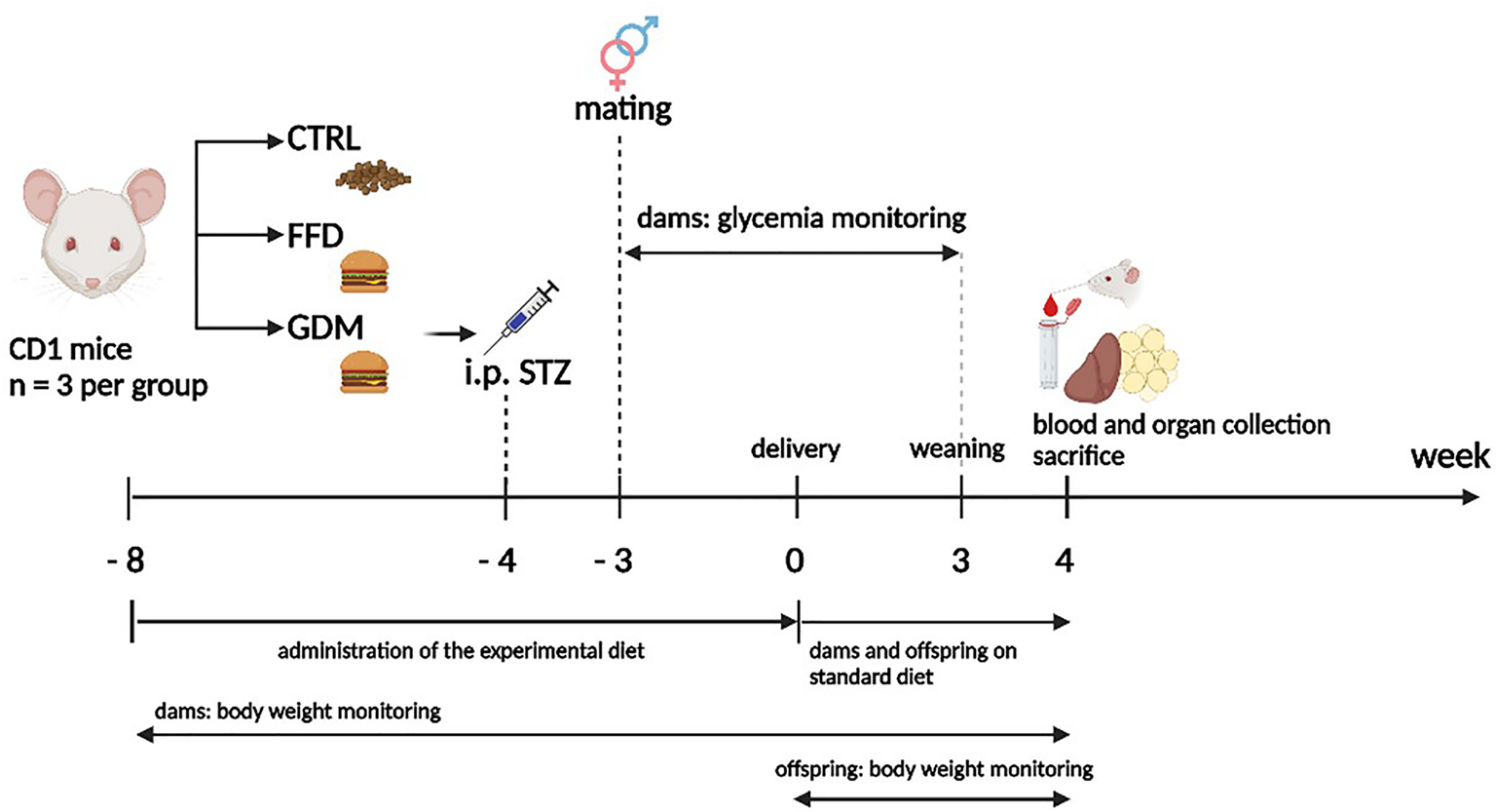
Flowchart of the experiment CTRL, dams on control diet; *FFD* dams consuming a fast-food diet, *GDM* dams on a fast-food diet with gestational diabetes mellitus, *STZ* streptozotocin; Created with https://BioRender.com.

The design of the experiment is depicted in Fig. 7. Female mice were divided into three groups of approximately equal body weight at the age of seven weeks. The control group (CTRL = 3) received a standard diet (296 kcal/100 g, 3% fat w/w, 11% energy derived from fat; Eypy-KMK20, Sedliště, Czech Republic) throughout the experiment. The FFD and GDM groups (n = 3, each) were administered conventional cheeseburgers^39^ (259 kcal/100 g, 11% fat w/w, 38% of energy derived from fat) untill parturition. The cheeseburgers were purchased from a local restaurant, ground, formed into homogeneous chunks weighing approximately 50 g, and stored at − 20 °C^39^. Five weeks after randomization, GDM females were administered STZ. Six weeks after randomization, female mice were mated with males fed a standard diet and housed together in a 1:1 ratio for five days. The presence of a vaginal plug was considered as gestation Day 0. The day of birth was considered postnatal day (PND) 0. If possible, litters were reduced to 4 females and 4 males per dam within 24 h from birth to assure uniformity in maternal care.

### Induction of gestational diabetes mellitus

A chemical-nutritional model of GDM was employed to mimic the pathophysiological state of human GDM. During the 5th week after the induction of the HFD, 3 females were fasted for 3 h and administered streptozotocin (Sigma‒Aldrich, St. Louis, Missouri) i.p. (27 g needle, BD Microlance™, Becton, Dickinson and Co, Drogheda, Ireland) at a dose of 50 mg/kg in 0.1 mmol/L citrate buffer (pH 4.5) for three consecutive days starting on Tuesday. To avoid sudden hypoglycemia after streptozotocin administration, mice were offered a 10% sucrose solution to drink for the next 24 h. Control females received an equal volume of the citrate buffer. Random blood glucose during gestation > 12 mmol/L was considered GDM.

### Maternal studies

Dams were weighed before randomization into the groups and then weekly until mating. During pregnancy, the weight gain was monitored every other day until delivery. Thereafter, the dams were weighed weekly until sacrifice. Chow consumption was monitored twice a week. Blood glucose concentrations were measured weekly using a glucometer (Accu-Chek Performa, Roche, Basel, Switzerland).

### Offspring studies

From the PND2, the offspring were weighed weekly. After weaning (PND 21), male and female offspring were separately caged (4–6 animals per cage) and administered a standard diet.

### Sacrifice and sample collection

Mice were sacrificed at PND28 using an overdose of i.p. administered anesthetics (100 mg/kg ketamine, Narkamon inj., Bioveta, Czech Republic; 10 mg/kg xylazine, Xylariem inj., Riemser, Germany). Before sacrifice, blood was collected from the retro-orbital plexus into heparin tubes. Organs were removed and weighed. Liver samples taken for total cholesterol and triacylglycerol analyses were weighed, and stored at − 80 °C for further analyses. Brown adipose tissue (BAT), perirenal (PRF), and perigonadal fat (PGF) tissues were dissected, weighed, and snap-frozen in liquid nitrogen. The relative weights of organs or fats to body weight were calculated.

### Extracellular DNA analyses

#### DNA isolation and quantification

Blood was centrifuged at 1600×*g* for 10 min at 4 °C, followed by the second centrifugation of 150 μL aliquots of the supernatant at 16,000×*g* for 10 min at 4 °C. DNA was isolated from plasma using the QIAamp DNA Blood Mini kit (Qiagen, Hilden, Germany). The isolated DNA was quantified using a Qubit 3.0 fluorometer and Qubit dsDNA high sensitivity assay (Thermo Fisher Scientific, Waltham, MA, USA).

#### Size profile of extracellular DNA

To overcome the problem of a low amount of DNA obtained from a single offspring, ecDNA samples from all offspring of one mother were pooled and concentrated using Concentrator plus (Eppendorf, Hamburg, Germany). Pooled samples were analyzed employing the 2100 Bioanalyzer Instrument using the Agilent High Sensitivity DNA Kit (Agilent Technologies, Santa Clara, California, USA) according to the manufacturer’s instructions. Briefly, 1 μL of each sample was used for on-chip electrophoresis and run for 45 min. A DNA standard ladder was used. The fragmentation profile is shown using a gel-like image generated by 2100 Expert Software (Agilent Technologies, Santa Clara, California, USA).

#### Nuclear and mitochondrial DNA

The fractions of nuclear and mitochondrial DNA were estimated using real-time PCR on the Mastercycler Realplex 4 (Eppendorf, Hamburg, Germany) and Sso Advanced Universal SYBR Green Supermix (Bio-Rad Laboratories, Hercules, CA, USA). A primer set for beta-2-microglobulin (B2m) was used to quantify nuclear DNA (F: 5′-CCCAGCTACTACCATCATTCAAGT-3′; R: 5′-GATGGTTTGGGAGATTG GTTGA TGT-3′), and a primer specific for the cytochrome B oxidase gene (cytB) was used to target mitochondrial DNA (F: 5′-TGTCAGATATGTCCTTCAGCAAGG-3′; R: 5′-TGCTAAACTCTGCAGGCGTAT-3′). The PCR program was adjusted according to the protocol of an employed Mastermix as follows: 1 cycle of 5 min at 95 °C followed by 40 cycles of 15 s at 94 °C for denaturation, annealing for 30 s at 60 °C, extension for 30 s at 72 °C, and final extension for 5 min at 72 °C. The ecDNA of both nuclear and mitochondrial origin was expressed in genome equivalents (GE) per ml of plasma.

### Deoxyribonuclease activity

Nonspecific deoxyribonuclease (DNase) activity was determined using a single radial enzyme dispersion assay (SRED)^64^ with the green-fluorescent dye GoodView™ (SBS Genetech, Beijing, China). Samples were loaded onto a 1% agarose gel containing 0.5 M Tris–HCl, 10 mM CaCl_2_, 10 mM MgCl_2_, pH 7.5, and 0.5 mg/mL DNA isolated from chicken livers. The gel with loaded samples was incubated at 37 °C for 17 h in the dark. Dilutions of DNase I (QIAGEN GmbH, Hilden, Germany) were used to construct a calibration curve. Diameters of circles of hydrolyzed DNA (reflecting DNase activity) were measured using ImageJ software (NIH, Maryland, USA).

### Biochemical analyses

All biochemical analyses were conducted in blood plasma after centrifugation at 1600×*g* centrifugation for 10 min at 4 °C. Advanced oxidation protein products (AOPP) measurement was performed via the spectrophotometric method described previously^55^. The concentrations of cytokines (IL-1α, IL-6, IL-17A, IL-23, IL-10, TNF-α, GM-CSF, MCP-1, and INF-β) in plasma were analyzed using the LEGENDplex™ Mouse Inflammation Panel (13-plex) in V-bottom plates (Biolegend, San Diego, California, USA) and measured on a DxFlex cytometer (Beckman, Indianapolis, Indiana, USA) following the manufacturer’s instructions. Cytokine concentrations were calculated from the calibration curves of the standard solutions. Creatinine, high-density lipoprotein cholesterol (HDL-C) and low-density lipoprotein cholesterol (LDL-C) concentrations, and the activity of liver enzymes (aspartate aminotransferase—AST, alanine aminotransferase—ALT, alkaline phosphatase—ALP) were determined using the Biolis 24i Premium analyzer (Tokyo Boeki Machinery, Tokyo, Japan) due to the low volume of plasma only in thirty-three offspring. The concentrations of total cholesterol and triacylglycerols (TAG) in the liver tissue were determined by the modified spectrophotometrical methods of Abell et al.^65^ and Jover^66^, respectively. The accumulation of cholesterol and TAG was expressed in mmol per kg of liver tissue.

### Statistical analysis

Data were evaluated using GraphPad Prism 9 statistical software (GraphPad Software Inc., USA). The body weight gains of dams and offspring were evaluated using repeated measures of two-factor analysis of variance (ANOVA). Other data were analyzed using two-factor ANOVA with diet and sex as fixed factors, followed by Sidak’s post hoc test for multiple comparisons, except for the relative weights of testes and uteri, which were analyzed using one-way ANOVA. Pearsons’s correlation coefficients were calculated. Cytokine data were logarithmically transformed before analyses, as they were nonnormally distributed. Data are presented as the mean and standard deviation (SD). Data on extracellular DNA and its components are presented as the mean and standard error of measurement (SEM). P values < 0.05 were considered statistically significant.

The partial least squares-discriminant analysis (PLS-DA) model using the Simca v.16 software (Sartorius Stedim Data Analytics AB, Umea, Sweden) was employed to identify the set of variables with the largest discriminatory power between the groups of control males and females, or FFD and GDM offspring. For the detection of outliers and grouping, principal component analysis (PCA) was used. Bodyweight, the relative weight of BAT, PRF, kidneys, and liver, the concentrations of AOPPs, ecDNA, IL-1α, IL-6, IL-17A, IL-23, IL-10, TNF-α, GM-CSF, MCP-1, and INF-β, the concentrations of ncDNA and mtDNA, the activity of DNase, and the liver cholesterol and TAG content were entered as explanatory variables into the PLS-DA model. Before modeling, variables with high skewness and a low min-to-max ratio were logarithmically transformed and all data were mean-centered. Variables with a variable of importance for the projection (VIP) value ≥ 1.00 were considered important between-group discriminants. A post-hoc power test was run for variables assigned by PLS-DA as significant contributors to between-group separation in FFD and GDM males (clincalc.com, ClinCalc.LLC, Arlington Heights, IL, USA; accessed on 9 June 2021).

### Institutional review board statement

This study was conducted in compliance with the EU Guidelines for Scientific Experimentation on Animals and according to the protocol approved by The State Veterinary and Food Administration of the Slovak Republic under protocol code 2606/16-221.

## Supplementary Information

Supplementary Information.

## Data Availability

The datasets generated and/or analyzed during the current study are available in the [FIGSHARE] repository, (https://doi.org/10.6084/m9.figshare.20436627).
