# Supplementary material for: Severe gestational diabetes mellitus in lean dams is associated with low IL-1α levels and affects the growth of the juvenile mouse offspring
Source: Sci Rep. 2023 Jan 30;13:1700. doi: 10.1038/s41598-023-28903-7 (PMC9886986; doi:10.1038/s41598-023-28903-7)
Supplement: Supplementary file 1 — Supplementary Information. [file 41598_2023_28903_MOESM1_ESM.pdf]

Severe gestational diabetes mellitus in lean dams is associated with low IL-1α levels and affects the growth of the juvenile mouse offspring

Lucia Mihalovičová[1,7], Veronika Kunšteková[1,2,7], Dávid Miláček[1], Jakub Janko[1], Michal Pastorek[1], Barbora Konečná[1], Radana Gurecká[1,3], Zuzana Rausová[4], Oľga Uličná[4], Peter Celec[1,5,6], and Katarína Šebeková[1,*]

[1]Institute of Molecular Biomedicine, Faculty of Medicine, Comenius University, 81108 Bratislava, Slovakia
[2]Department of Biology, Faculty of Medicine, Slovak Medical University, 833 03 Bratislava, Slovakia
[3]Institute of Medical Physics, Biophysics, Informatics and Telemedicine, Faculty of Medicine, Comenius University, 81108 Bratislava, Slovakia
[4]Pharmacobiochemical Laboratory of 3rd Department of Internal Medicine, Faculty of Medicine, Comenius University, 81108 Bratislava, Slovakia
[5]Institute of Pathophysiology, Faculty of Medicine, Comenius University, 811 08 Bratislava, Slovakia
[6]Department of Molecular Biology, Faculty of Natural Sciences, Comenius University, 842 15 Bratislava, Slovakia
[7]These authors contributed equally to this work

Supplementary Table S1. Biochemical data of the offspring

	MALES						FEMALES					
	CTRL (N=5)		FFD (N=6)		GDM (N=10)		CTRL (N=5)		FFD (N=5)		GDM (N=2)	
	M	SD	M	SD	M	SD	M	SD	M	SD	M	SD
**Creat. [µmol/L]**	16.3	±2.9	17.4	±6.0	14.7	±2.5	18.3	±2.2	14.4	±3.0	18.0	±1.8
**AST [µkat/L]**	0.54	±0.09	0.64	±0.36	0.72	±0.16	0.67	±0.12	0.65	±0.07	0.61	±0.04
**ALT [µkat/L]**	0.21	±0.03	0.26	±0.05	0.28	±0.06	0.24	±0.01	0.22	±0.05	0.18	±0.02
**ALP [µkat/L]**	1.88	±0.17	2.60	±0.29	2.89	±0.46	2.8	±0.17	3.11	±0.30	2.77	±0.42
**HDL-C [mmol/L]**	1.98	±0.17	2.60	±0.11	1.98	±0.15	1.41	±0.23	1.36	±0.17	1.50	±0.53
**LDL-C [mmol/L]**	0.67	±0.06	0.63	±0.08	0.76	±0.11	0.57	±0.10	0.54	±0.12	0.65	±0.18

CTRL, offspring of dams consuming a control diet; FFD, offspring of dams administered a fast-food diet (cheeseburgers); GDM, offspring of dams with gestational diabetes mellitus; Creat., plasma creatinine; AST, plasma aspartate aminotransferase activity; ALT, plasma alanine aminotransferase activity; ALP, plasma alkaline phosphatase activity; HDL-C, high-density lipoprotein cholesterol concentration in plasma; LDL-C, low-density lipoprotein cholesterol concentration in plasma; M, mean; SD, standard deviation; N, number of animals.

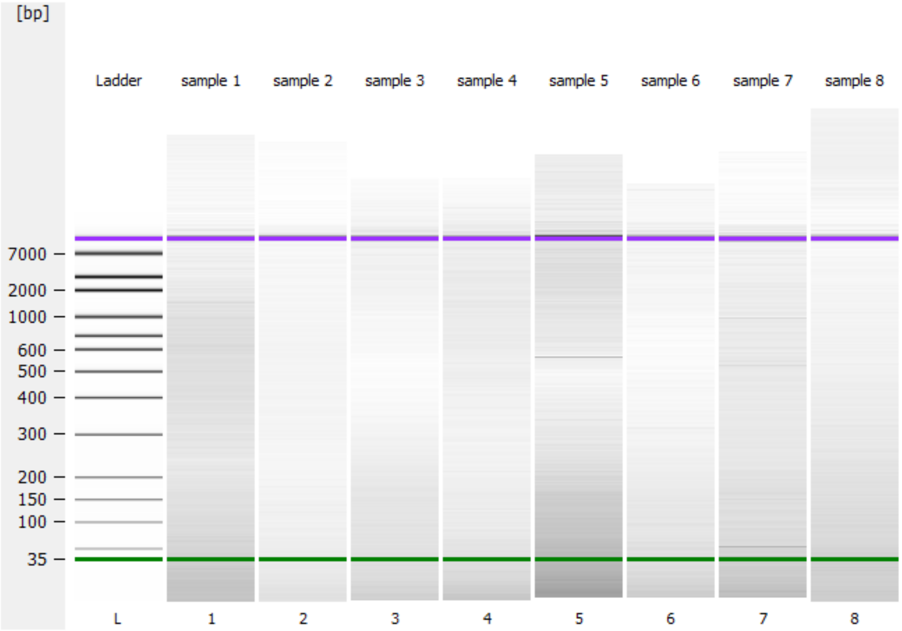

Supplementary Figure S1. Fragmentation profiles of extracellular deoxyribonucleic acid (ecDNA) of the offspring.

bp: base-pairs; samples 1,2,3 – pooled ecDNA from offspring of three dams consuming a standard diet; samples 4, 5 – pooled ecDNA of offspring of two dams with gestational diabetes mellitus; samples 6, 7, 8 – pooled ecDNA from offspring of three dams consuming a fast-food diet.
